# Supplementary material for: Giant Rashba splitting in PtTe/PtTe2 heterostructure
Source: Nat Commun. 2025 Mar 18;16:2667. doi: 10.1038/s41467-025-57835-1 (PMC11920107; doi:10.1038/s41467-025-57835-1)
Supplement: Supplementary file 1 — Supplementary Information [file 41467_2025_57835_MOESM1_ESM.pdf]

Supplementary Information for

Giant Rashba splitting in PtTe/PtTe$_2$ heterostructure

Runfa Feng[1], Yang Zhang[1], Jiaheng Li[1], Qian Li[1], Changhua Bao[1], Hongyun Zhang[1,2], Wanying Chen[1], Xiao Tang[1], Ken Yaegashi[2], Katsuaki Sugawara[2,3], Takafumi Sato[2,3], Wenhui Duan[1,4,5], Pu Yu[1,4,*] & Shuyun Zhou[1,4,*]

[1] *State Key Laboratory of Low-Dimensional Quantum Physics and Department of Physics, Tsinghua University, Beijing 100084, People's Republic of China*

[2] *Advanced Institute for Materials Research (WPI-AIMR), Tohoku University, Sendai 980-8577, Japan*

[3] *Department of Physics, Graduate School of Science, Tohoku University, Sendai, 980-8578, Japan*

[4] *Frontier Science Center for Quantum Information, Beijing 100084, People's Republic of China*

[5] *Institute for Advanced Study, Tsinghua University, Beijing 100084, People's Republic of China*

** Correspondence should be sent to yupu@mail.tsinghua.edu.cn, syzhou@mail.tsinghua.edu.cn.*

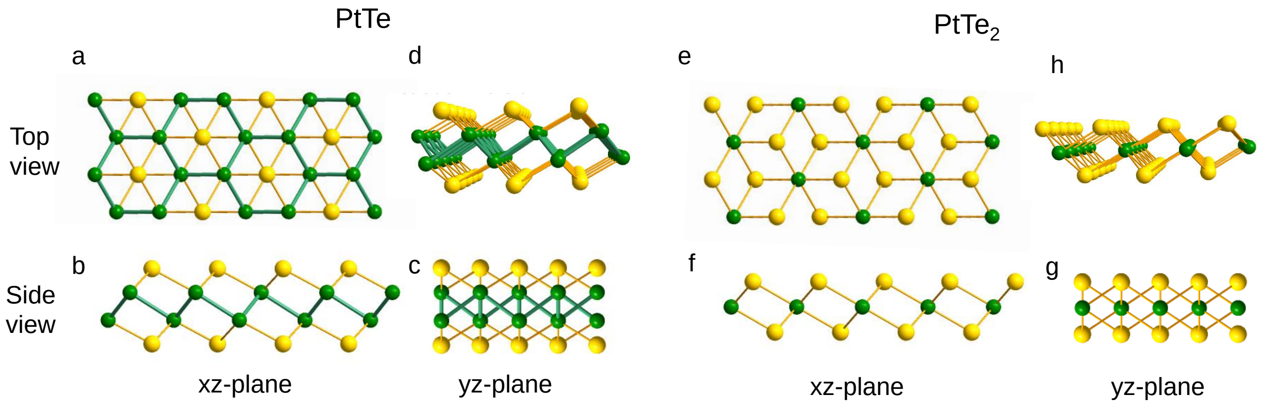

Supplementary Figure 1| Crystal struture of the PtTe and PtTe$_2$. **a**, Top view of PtTe along z direction. **b**, Side view of PtTe along y direction. **c**, Side view of PtTe along x direction. **d**,3D view of PtTe. **e-g**, The same as (**a-d**) but for PtTe$_2$.

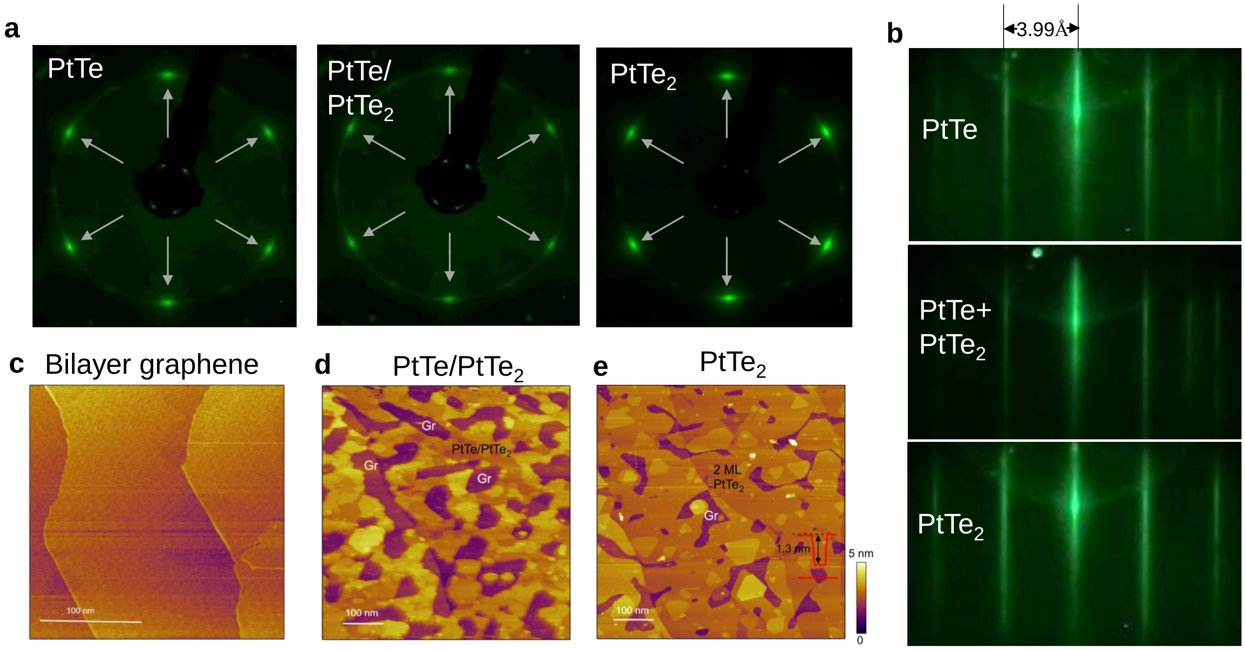

Supplementary Figure 2| Characterization of the PtTe, PtTe/PtTe$_2$ and PtTe$_2$ films. a,b, Low energy electron diffraction (LEED) (**a**) and reflection high energy electron diffraction (RHEED) pattern (**b**) of PtTe, PtTe/PtTe$_2$ and PtTe$_2$ films. The sharp LEED spots and RHEED stripes indicate high crystallization quality. **c-e**, Scanning tunneling microscope (STM) image of bilayer graphene/SiC substrate (**c**), PtTe/PtTe$_2$ (**d**) and PtTe$_2$ (**e**). The results indicate the presence of atomically flat surface morphology among all samples.

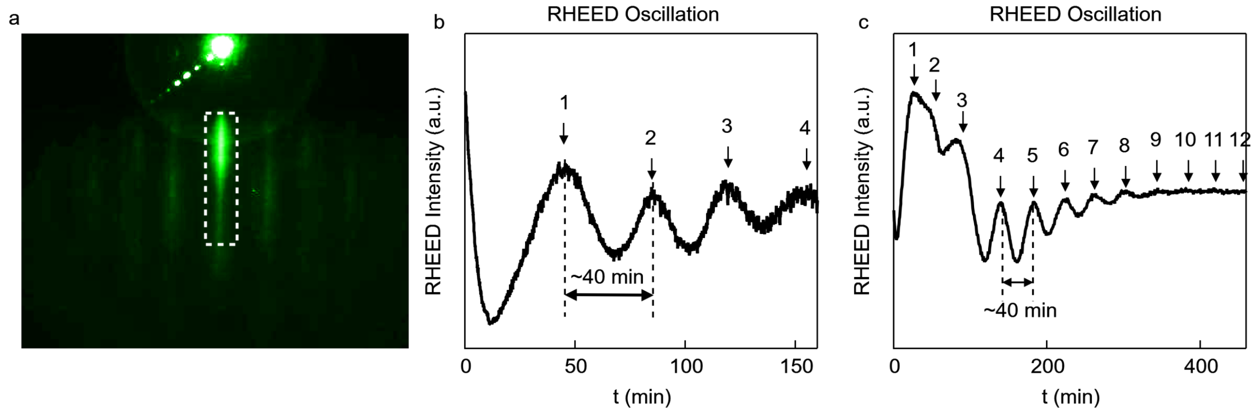

Supplementary Figure 3| Growth rate determined by RHEED oscillation. **a**, RHEED image of a 4 ML $PtTe_2$ film. The white broken box shows the area where RHEED intensity integrated.. **b**, RHEED oscillations of the 4 ML $PtTe_2$ during growth with the growth rate of 40 minutes per layer. **c**, Another RHEED oscillation of 12 ML $PtTe_2$ (namely, $(PtTe_2)_{2N}$ with N = 6), where the growth rate is also around 40 minutes per layer.

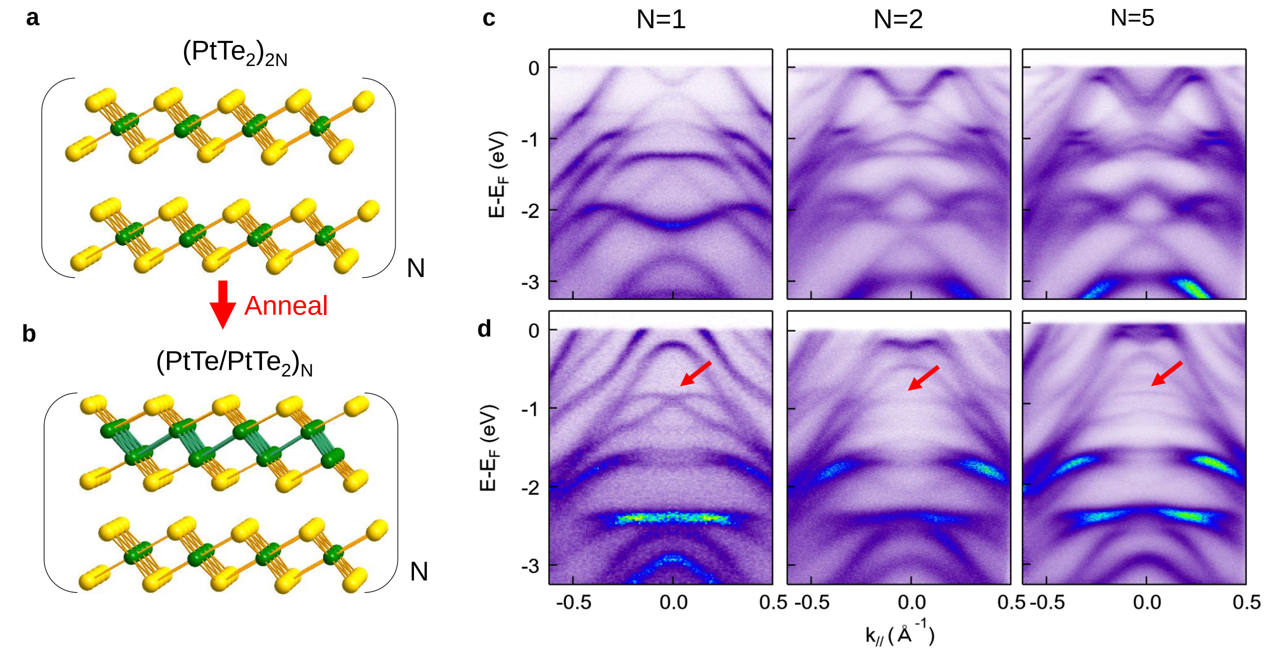

Supplementary Figure 4| Band structure of PtTe/PtTe$_2$ films with different thickness. a, Schematic structure of (PtTe$_2$)$_2N$. **b**, Schematic structure of (PtTe/PtTe$_2$)$_N$. **c**, ARPES dispersion image measured near Γ point in as-grown (PtTe$_2$)$_2N$ films. **d**, ARPES dispersion image measured near Γ point in annealing (PtTe/PtTe$_2$)$_N$ films. Rashba band only appears in N = 1 film.

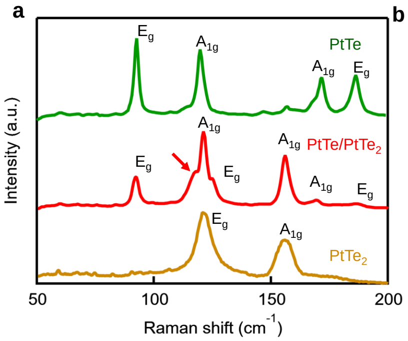

b

PtTe$_2$		PtTe		PtTe/PtTe$_2$	
Cal	Exp	Cal	Exp	Cal	Exp
109 (E_g)	121	93 (E_g)	92	91 (E_g)	92
158 (A_{1g})	156	123 (A_{1g})	121	120 (A_{1g})	121
		184 (A_{1g})	171	124 (E_g)	125
		188 (E_g)	186	161 (A_{1g})	156
				176 (A_{1g})	171
				184 (E_g)	187

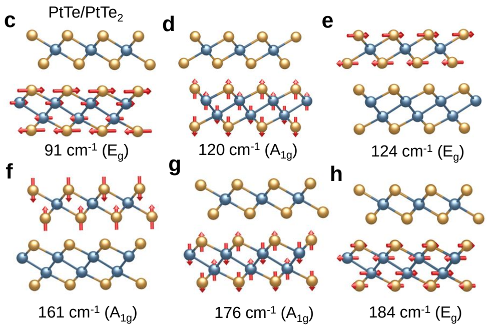

Supplementary Figure 5| Experimental and calculated Raman spectra for PtTe$_2$, PtTe/PtTe$_2$ and PtTe. a, Experimental Raman spectra. **b**, Comparison of calculated and experimental Raman spectra. The unit is cm^{-1}. **c-h**, Calculated Raman modes in PtTe/PtTe$_2$ heterostructure.

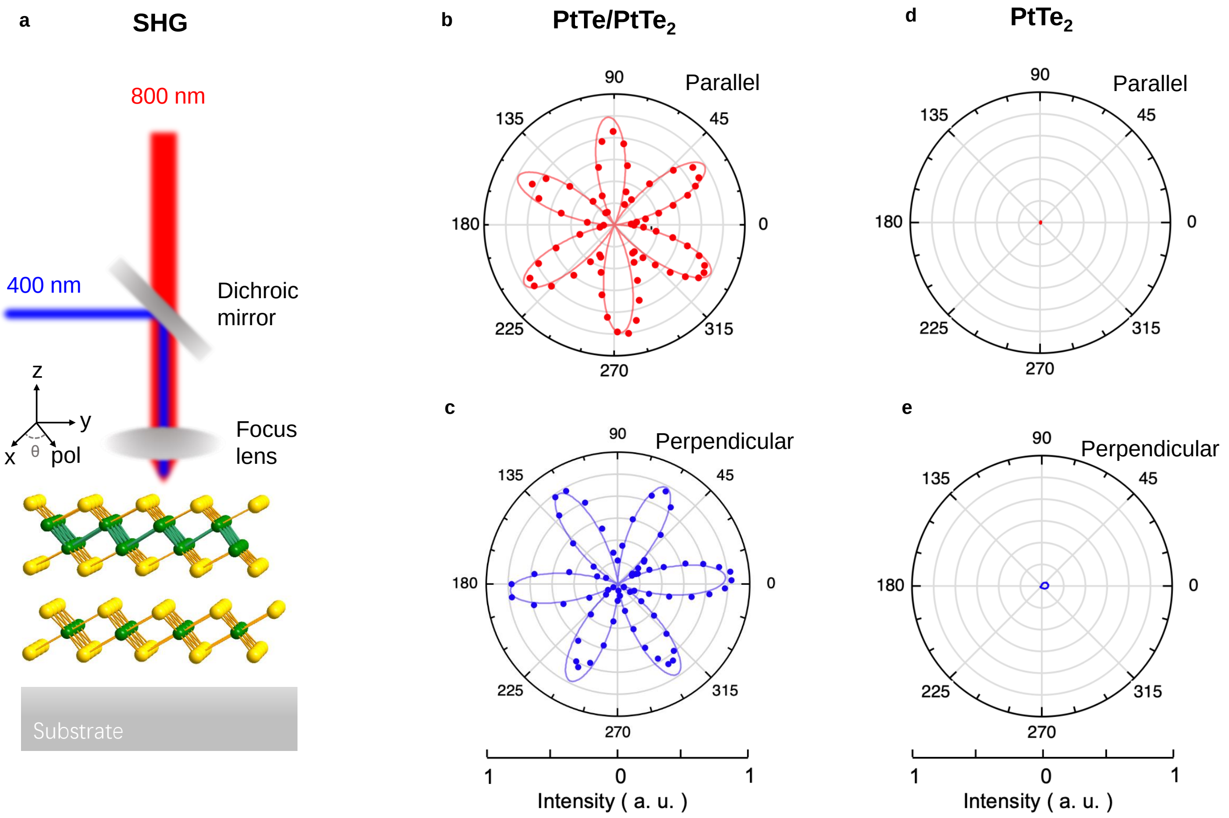

Supplementary Figure 6| Inversion symmetry breaking in PtTe/$PtTe_2$ heterostructure revealed by SHG. a, Schematic illustration for SHG measurements. **b,c**, Rotational anisotropy SHG pattern of PtTe/$PtTe_2$ heterostructure consisting of 1 ML PtTe and 1 ML $PtTe_2$, with the polarization of SHG signal parallel (**b**) and perpendicular (**c**) to the incident laser. **d,e**, Corresponding data to (**b,c**) but for bilayer $PtTe_2$, where negligible SHG signal is observed.

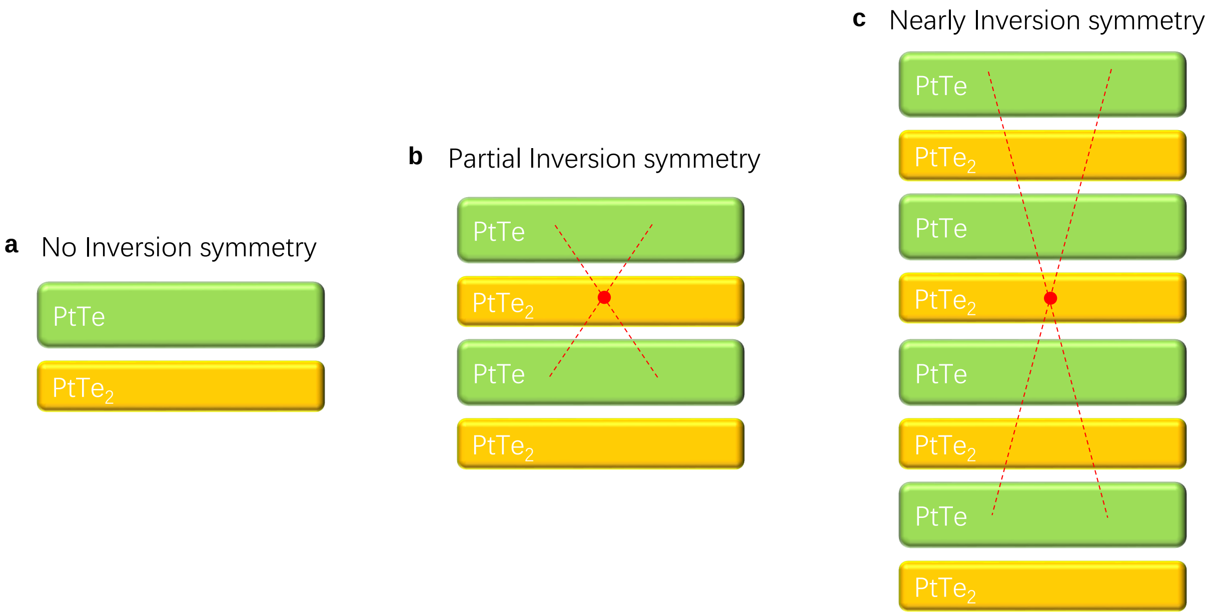

Supplementary Figure 7|Inversion symmetry analysis for PtTe/$PtTe_2$ heterostructure with different thickness. **a**, Schematic illustration for $(PtTe/PtTe_2)_1$, where inversion center is missing. **b**, Schematic illustration for $(PtTe/PtTe_2)_2$, where inversion center could be found in the top three layers. This result suggest the reduced polarity. **c**, Schematic illustration for $(PtTe/PtTe_2)_4$, the inversion symmetry nearly recovers.

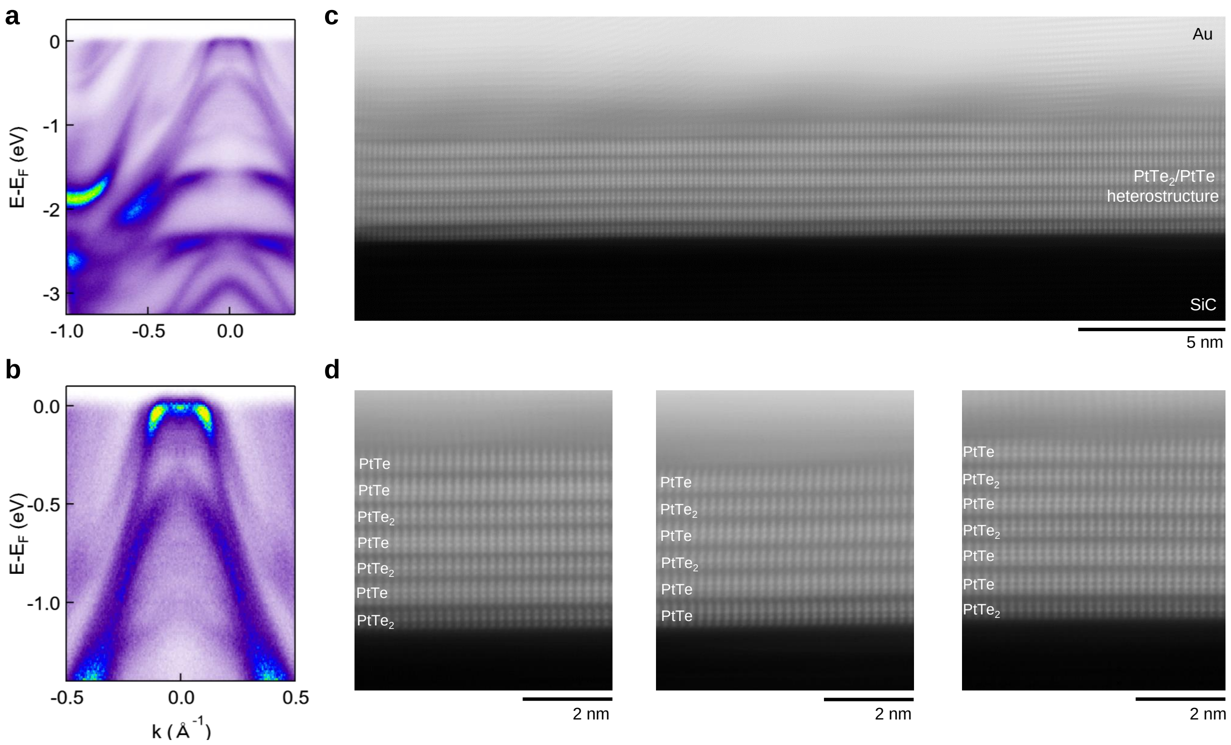

Supplementary Figure 8|Multilayer heterostructure with different stacking of PtTe and $PtTe_2$. **a,b**, ARPES dispersion image of multilayer PtTe/$PtTe_2$ films along ΓM direction (**a**) and zoom-in dispersion near Fermi level (**b**). The sample was obtained by annealing a ten-layer $PtTe_2$ film. **c**, STEM image of multilayer PtTe/$PtTe_2$ on graphene/SiC substrates. **d**, Zoom-in STEM image to resolve the stacking sequence of PtTe and $PtTe_2$ layers, In all three images, we observe the presence of stacking faults in various layers with continuously stacked PtTe bilayers.

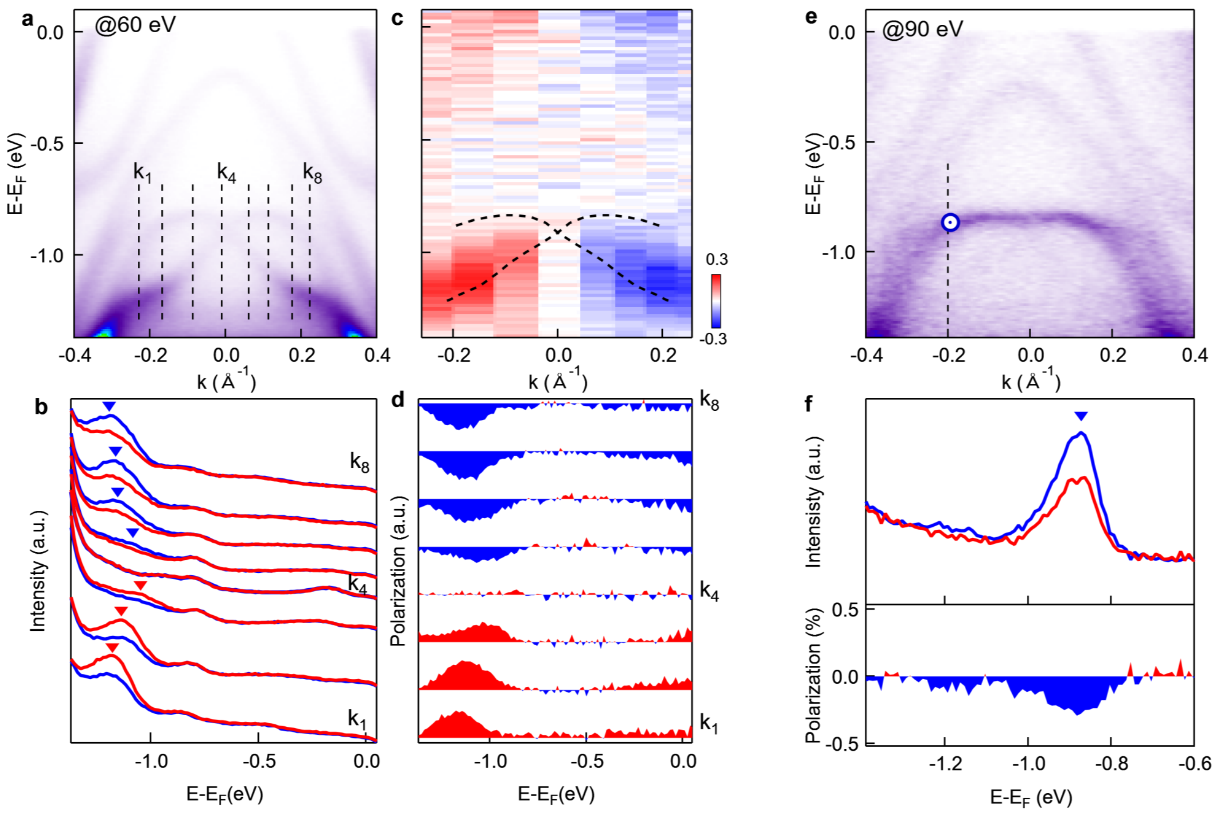

Supplementary Figure 9| Distinct ARPES intensity using different photon energy due to matrix element effect. a, ARPES dispersion image of PtTe/PtTe$_2$ films with photon energy of 60 eV. The ARPES intensity of lower Rashba-splitting band was enhanced due to matrix element effect at this photon energy. Black broken lines mark the position for energy distribution curves (EDCs) shown in (**b**). **b**, The spin-resolved EDCs marked as dash lines in (**a**). **c**, The spin-polarization image of Spin-ARPES with strong spin polarization in lower Rashba-splitting band. **d**, The spin-contrast EDCs of Spin-ARPES. **e**, ARPES dispersion image of PtTe/PtTe$_2$ films with photon energy of 90 eV, where intensity of upper Rashba-splitting band was enhanced due to matrix element effect. **f**, The spin-resolved EDCs with strong spin polarization in upper Rashba-splitting band.

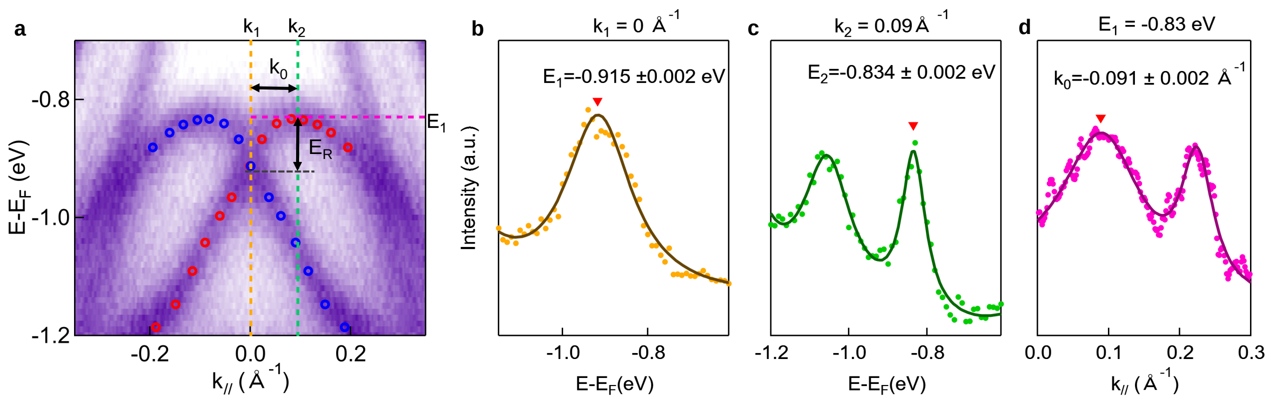

Supplementary Figure 10| Zoom-in dispersion to extract the Rashba coefficient. **a**, Zoom-in band dispersion of the Rashba-splitting bands. **b**, Energy distribution curve (EDC) at Γ point. **c**, EDC at top of the valence band. **d**, Momentum distribution curve (MDC) at top of the valence band. All the curves were fitted by Lorentzian functions.

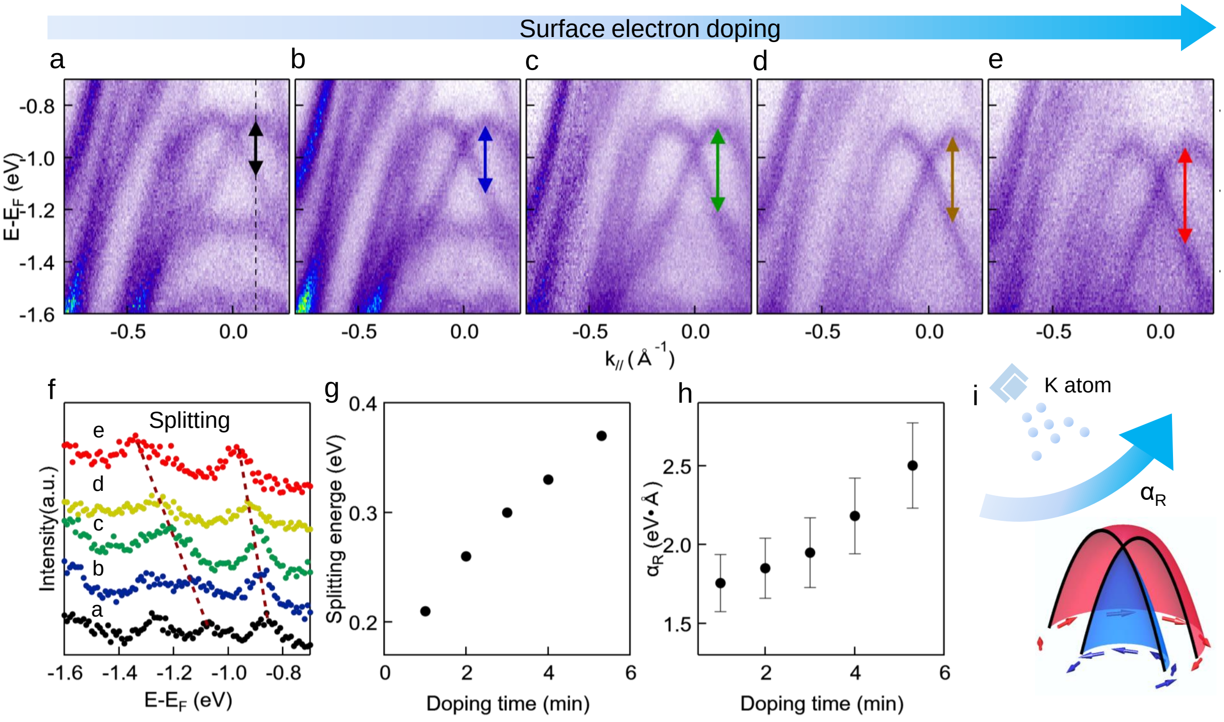

Supplementary Figure 11|Enhanced Rashba splitting upon surface electron doping. a-e, Evolution of dispersion images upon surface electron doping via K deposition. The arrows mark the energy separation of the spin splitting at that momentum. **f**, Energy distribution curves measured at the same momentum as indicated in (**a**). **g**, Splitting energy evolves with doping obtained at k = 0.1 $\AA^{-1}$ from (**a-e**). **h**, Extracted Rashba coefficient α_R at different doping time. Error bars are obtained from fitting errors. **i**, Schematic illustration of enhanced Rashba splitting upon surface electron doping via K deposition.

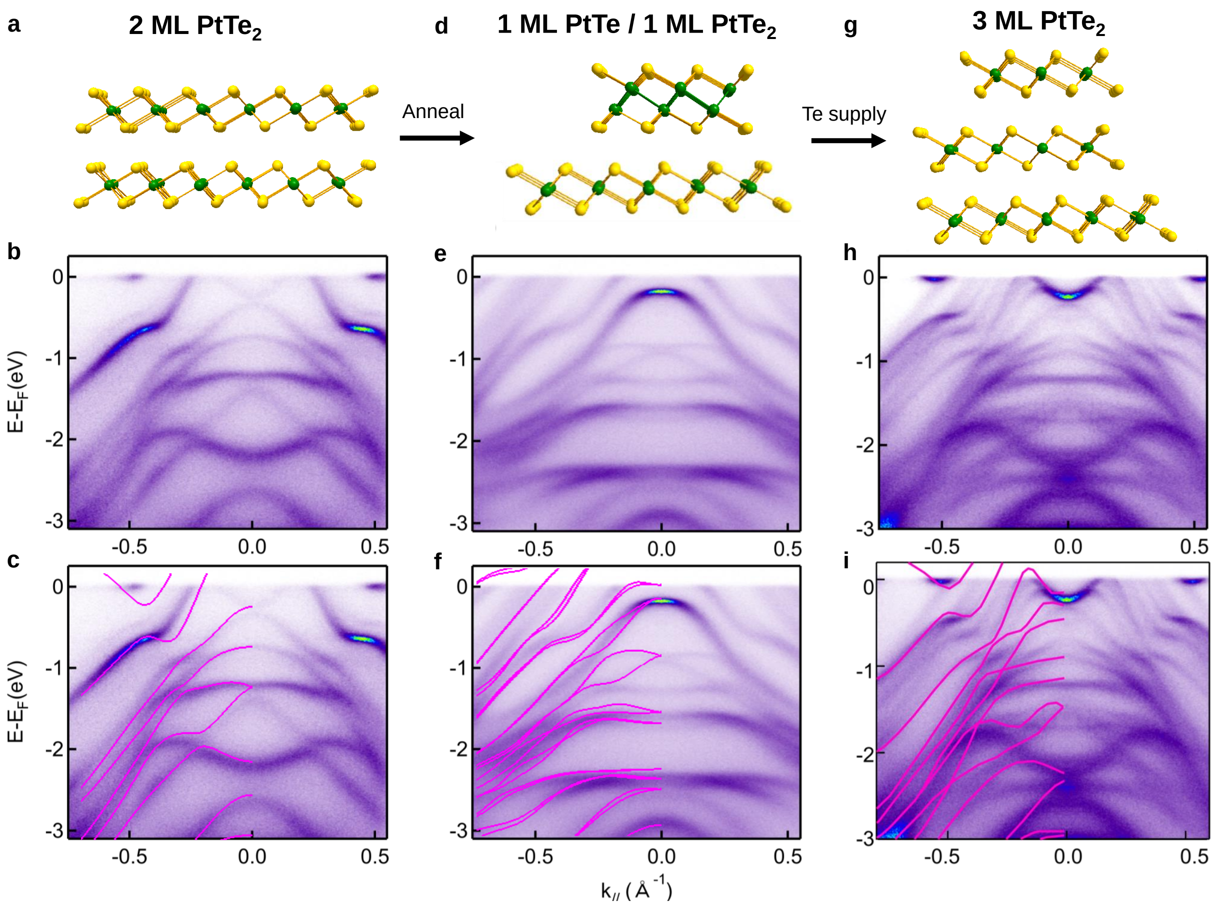

Supplementary Figure 12| Structural transition between PtTe and $PtTe_2$ in the heterostructure. **a**, Schematic structure of bilayer $PtTe_2$. **b**, ARPES dispersion image measured along the ΓM direction in as-grown bilayer $PtTe_2$ films. **c**, The same as (**b**) but with the superposed calculated bilayer $PtTe_2$ band structure (pink line). **d-f**, The same as (**a-c**) but for PtTe/$PtTe_2$ obtained after annealing in UHV. The superposed calculated PtTe/$PtTe_2$ band structure (pink line) is shown in (**f**). **g-i**, The same as (**d-f**) but for multilayer $PtTe_2$ obtained after subsequent annealing in Te flux The calculated band structure for 3 ML $PtTe_2$ (pink line) were superposed on (**i**), which shows excellent consistency with the experimental results, verifying the stacking sequence and thickness of the formed sample.

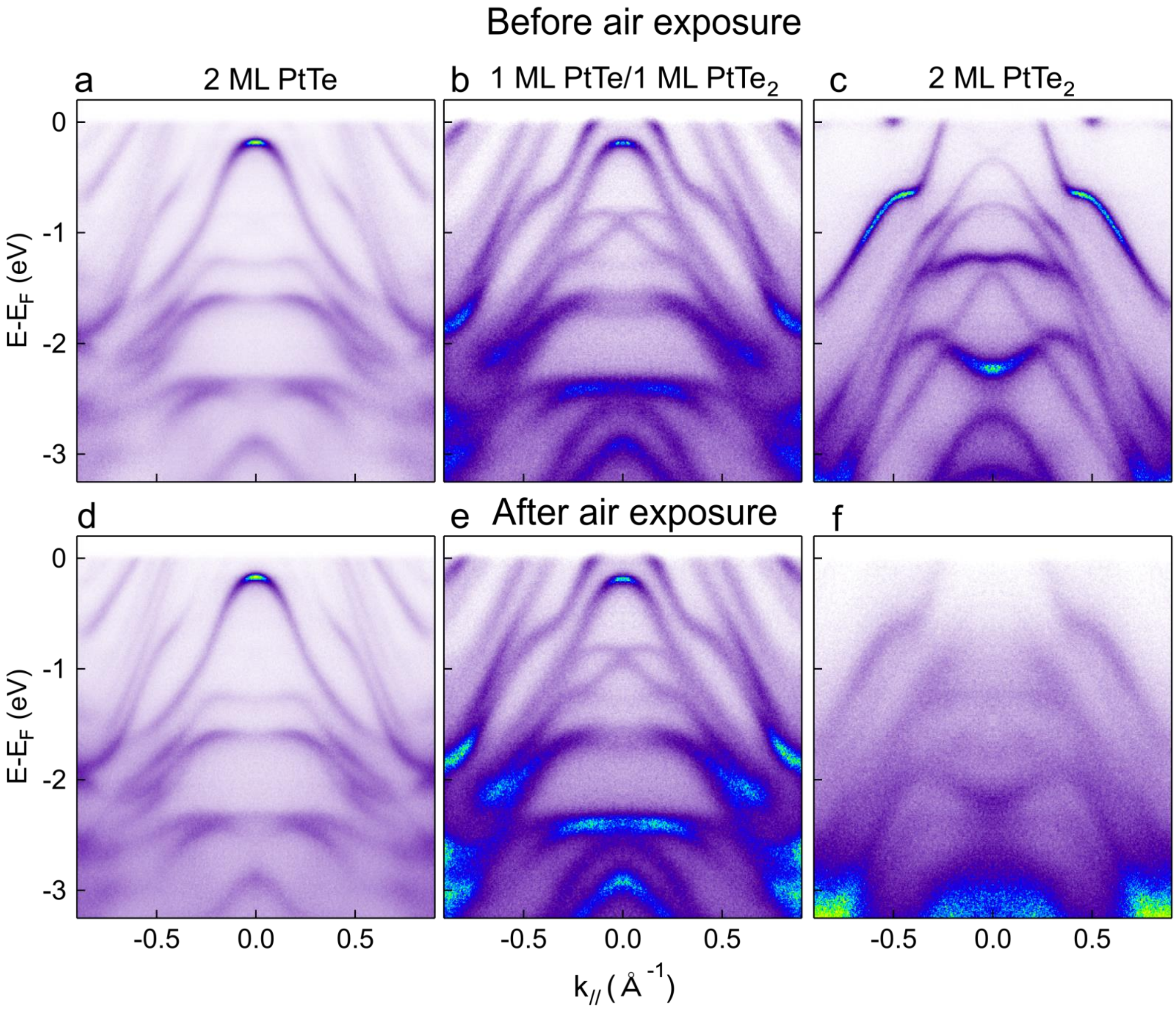

Supplementary Figure 13| Stability test of $PtTe_2$, $PtTe/PtTe_2$ and PtTe samples. **a-c**, Data acquired *in situ* after the film growth for 2 ML PtTe (**a**), $PtTe/PtTe_2$ (**b**) and 2 ML $PtTe_2$ (**C**). **d-f**, Data acquired after air exposure for two days. The PtTe (**d**) and $PtTe/PtTe_2$ (**e**) samples show a notable improved stability as comparing with that of $PtTe_2$ (**f**).
